# The Characterization of OXA-232 Carbapenemase-Producing ST437 *Klebsiella pneumoniae* in China

**DOI:** 10.1155/2020/5626503

**Published:** 2020-07-08

**Authors:** Xingbei Weng, Qiucheng Shi, Sheng Wang, Yubo Shi, Dinghe Sun, Yunsong Yu

**Affiliations:** ^1^Department of Laboratory Medicine, Ningbo Hospital, Zhejiang University School of Medicine, Ningbo, China; ^2^Department of Laboratory Medicine, Ningbo First Hospital, Ningbo, China; ^3^Department of Infectious Diseases, Sir Run Run Shaw Hospital, Zhejiang University School of Medicine, Hangzhou, China; ^4^Key Laboratory of Microbial Technology and Bioinformatics of Zhejiang Province, Hangzhou, China

## Abstract

Carbapenem-resistant *Klebsiella pneumoniae* (CRKP) was epidemic around the world and become a global threat to public health. The most important carbapenem-resistant mechanism is producing carbapenemases, especially *Klebsiella pneumoniae* carbapenemase (KPC), which is prevalent in the international clonal complex CC11. The high-risk multidrug-resistant CC11 is widespread worldwide, and KPC-producing and (New Delhi metallo) NDM-producing strains had been reported in this clonal complex before; moreover, cases with the CC11 strain faced more severe forms of drug resistance and treatment challenges than other clonal complexes. In this study, we identified an OXA-232-producing ST437 *Klebsiella pneumoniae* isolate in China, which belonged to CC11. The isolate was resistant to *β*-lactams, aminoglycosides, and fluoroquinolones but susceptible to fosfomycin, tigecycline, and colistin. The *bla*_OXA-232_ gene was located on a 6141 bp ColKP3-type nonconjugative plasmid, and the plasmid was transformed by chemical transformation successfully. This is the first report of OXA-232-producing ST437 *K. pneumoniae* in China, a new clone of high-risk multidrug-resistant CC11.

## 1. Introduction

With the spread of carbapenemase genes, the emergence and dissemination of carbapenem-resistant *Klebsiella pneumoniae* (CRKP) is an escalating global threat. The most important carbapenem-resistant mechanism is producing carbapenemases, which includes KPC, NDM, VIM, IMP, and OXA-48-like. A nationwide survey conducted in China showed that acquisition of two carbapenemase genes, *bla*_KPC-2_ and *bla*_NDM_, was responsible for phenotypic resistance in 90% of the CRE strains tested (58% and 32%, respectively), among which several major strain types, such as ST11 of *K. pneumoniae*, were identified [1]. Currently, local clonal disseminations of OXA-48-type carbapenemases in China have been reported [2–4], but we are short of precise data concerning the epidemiology of this carbapenemase.

OXA-48-type carbapenemases were first identified in 2001 [5] and characterized by low level of carbapenem resistance. To date, 11 OXA-48-type carbapenemases have been described: OXA-48, OXA-162, OXA-181, OXA-204, OXA-232, OXA-244, OXA-245, OXA-247, OXA-436, OXA-484, and OXA-519. But OXA-163 and OXA-405 hydrolyze the extended-spectrum cephalosporins and show limited activities against the carbapenems. Moreover, other OXA-48-variants such as OXA-252, OXA-438, OXA-439, OXA-505, OXA-517, OXA-566, and OXA-567 are listed only in GenBank, and it is currently uncertain if these enzymes have carbapenemase activities [6]. OXA-232 is part of OXA-48-type variants and firstly identified from three patients transferred from India to France in 2011, which differed from OXA-48 by five amino acid substitutions and was located on a 6141 bp ColE-type nonconjugative plasmid [7].

Clonal complex 11 (CC11) denotes the STs differed from ST11 by one housekeeper gene, including ST11, ST258, ST340, ST437, ST757, ST855, and others. CC11 is an epidemic *K. pneumoniae* clonal complex [1], and cases with the CC11 strain faced more severe forms of drug resistance and treatment challenges than other clonal complexes [8]. KPC-producing [1] and NDM-producing [9] CC11 have been reported in China, and no one reported OXA-48-type-producing CC11 clone in China although a clonal dissemination of OXA-48-producing ST147 and ST383 [4] as well as two clonal disseminations of OXA-232-producing ST15 have been reported in China [2, 3]. This is the first report of OXA-232-producing ST437 *K. pneumoniae* in China, a new clone of high-risk multidrug-resistant CC11. The aim of this study was to determine the antimicrobial resistance profile, and identify the carrier plasmid and the flanking region of the *bla*_OXA-232_ gene.

## 2. Materials and Methods

### 2.1. Isolation and Identification

*K. pneumoniae* NB5306 was recovered from a blood culture from a 72-year-old female patient. The patient hospitalized (Day 0) in hematology ward in Ningbo First Hospital, China, with the history of aplastic anemia. In order to prevent nosocomial infection, ceftriaxone/sulbactam (3 g q8 h) was administrated after admission for 2 days, and then administration was changed to biapenem (0.3 g q8 h) for 10 days; the patient was stable and had no invasive procedure. However, the patient had a fever at Day 20, and the highest temperature was up to 40°C, with white blood cell count being 0.60 × 10^9^/L, neutrophil% 20.0%, and c-reactive protein (CRP) 37.20 mg/L. Blood culture was examined immediately on Day 20, and the empiric therapy was administrated with imipenem (0.5 g q6 h), vancomycin (0.5 g q12 h), and voriconazole (0.2 g q12 h). During the treatment, the temperature of patient was sustained, and her condition continued to deteriorate; therefore, the patient gave up further treatment on Day 22 and returned home. Unfortunately, we were informed that the patient died at home two days after discharge by phone follow-up. *K. pneumoniae* NB5306 was recovered from blood culture and was identified with VITEK® 2 automated microbial identification system.

### 2.2. Antimicrobial Susceptibility Testing

Antimicrobial susceptibility testing of MICs was performed by the agar dilution method or broth microdilution method (for tigecycline and colistin), with susceptibility defined according to Clinical and Laboratory Standards Institute (CLSI) (M100-S28) and European Committee on Antimicrobial Susceptibility Testing (EUCAST) (version 8.1, for tigecycline and colistin) guidelines.

### 2.3. Genome Sequencing and Bioinformatics Analysis

The genomic DNA was extracted by QIAamp DNA Mini Kit (Qiagen, Germany) and sent to Zhejiang Tianke Hi-tech Development Co., Ltd. (Tianke, Hangzhou, China) for library preparation and genome sequencing. Genome was sequenced by HiSeq instrument (Illumina, San Diego, CA, USA). Reads were *de novo* assembled using CLC Genomics Workbench (version 9.5.1). Multilocus sequence typing (MLST) was identified by the genome sequence using MLST 2.0 (https://cge.cbs.dtu.dk/services/MLST/). The antimicrobial resistome was identified using ResFinder 3.1 (https://cge.cbs.dtu.dk/services/ResFinder/). Mutations in two outer membrane porin-encoding genes *omp*K35 and *omp*K36 were determined using KX528043.1 and JX291114.1 as references, respectively.

### 2.4. Plasmid Conjugation and Chemical Transformation

Plasmid conjugation of the *bla*_OXA-232_ gene was attempted by conjugation experiments at 37°C using rifampicin-resistant *E. coli* EC600 or sodium azide-resistant *E. coli* J53 as recipient, and imipenem (0.12 mg/L) associated with rifampicin (700 mg/L) or sodium azide (300 mg/L) was used for selection.

The chemical transformation was performed when conjugation failed. Plasmid harboring *bla*_OXA-232_ gene was transferred into *E. coli* DH5*α* by chemical transformation with imipenem (0.12 mg/L) for selection. The MICs of NB5306-D (DH5*α* chemical transformant of NB5306) to ertapenem, meropenem, and imipenem were performed.

### 2.5. S1 PFGE and Southern Bolt

Genomic DNA was digested with S1-nuclease (Takara, Otsu, Japan) and electrophoresed on a PFGE system (Bio-Rad, Hercules, CA, USA) for 10 h at 14°C, with run conditions of 6 V/cm and pulse times from 2.16 s to 63.8 s. DNA fragments were transferred to a positively charged nylon membrane (Millipore, Billerica, MD, USA) and then hybridized with a digoxigenin-labelled *bla*_OXA-232_-specific probe. The fragments then were detected with an NBT/BCIP color detection kit (Roche, Mannheim, Germany).

### 2.6. The Primer Walking Sequencing

The plasmid containing *bla*_OXA-232_ was obtained by Axygen plasmid miniprep (Axygen Biosciences, USA), initial round of sequencing was from a known sequence at one end of the template, and initial primers were *bla*_OXA-48-_F (5′-ATGCGTGTATTAGCCTTATCGGCT-3′) and *bla*_OXA-48-_R (5′-CTAGGGAATAATTTTTTCCTGTTTGAG-3′); then, each subsequent round was initiated from a new primer, which is based on the end of the sequence obtained from the previous reaction. Finally, the full length of the plasmid was sequenced after some rounds of reactions. The replicon of plasmid was identified by plasmid finder 2.1 (https://cge.cbs.dtu.dk/services/PlasmidFinder/).

## 3. Results

### 3.1. Results of Antimicrobial Susceptibility Testing and MLST

NB5306 was resistant to ertapenem (MIC of 8 mg/L) but susceptible to imipenem (MIC of 0.25 mg/L), intermediate to meropenem (MIC of 2 mg/L). Moreover, NB5306 was susceptible to fosfomycin, tigecycline, and colistin but resistant to other *β*-lactams or *β*-lactams/*β*-lactamase inhibitor (cefotaxime, ceftriaxone, and piperacillin/tazobactam), aminoglycosides (gentamicin, tobramycin, and amikacin), as well as fluoroquinolones (ciprofloxacin and levofloxacin). NB5306 was identified as ST437 (*gap*A 3, *inf*B3, *md*h 1, *pgi* 1, *pho*E 1, *rpo*B 1, and *ton*B 31), with one housekeeper gene difference with ST11 (*gap*A 3, *inf*B3, *md*h 1, *pgi* 1, *pho*E 1, *rpo*B 1, and *ton*B 1).

### 3.2. Results of Antimicrobial Resistome

The antimicrobial resistome was comprised of genes conferring resistance to *β*-lactams (*bla*_OXA-232_, *bla*_CTX-M-55_, *bla*_TEM-1b_, and *bla*_SHV-11_), aminoglycosides (*aph* (3′)-IIa, *aph* (3″)-Ib, *aph* (6)-Id, and *rmt*B), fluoroquinolones (*oqx*A and *oqx*B), fosfomycin (*fos*A), trimethoprim (*dfr*A14), sulphonamide (*sul*2), phenicol (*flo*R), and tetracycline (*tet*A).

### 3.3. Results of Plasmid Conjugation and Chemical Transformation

NB5306 carried a wild-type *omp*K35 but a novel *omp*K36 variant, a highly conserved domain within the region of loop 3 (DVLPEFGGDTDTYGFDNFLQSRA NGV).

In the conjugation experiments, *bla*_OXA-232_ was unsuccessfully transferred to EC600 and J53. MICs of NB5306-D (DH5*α* chemical transformant of NB5306) to ertapenem, meropenem, and imipenem rose to 1 mg/L, 0.12 mg/L, and 0.5 mg/L, respectively, which was higher than the MICs of *E. coli* DH5*α* (ertapenem 0.03 mg/L, meropenem 0.03 mg/L, and imipenem 0.06 mg/L, respectively) (Table 1).

### 3.4. Results of S1 PFGE and Plasmid Sequencing

**Figure 1:**
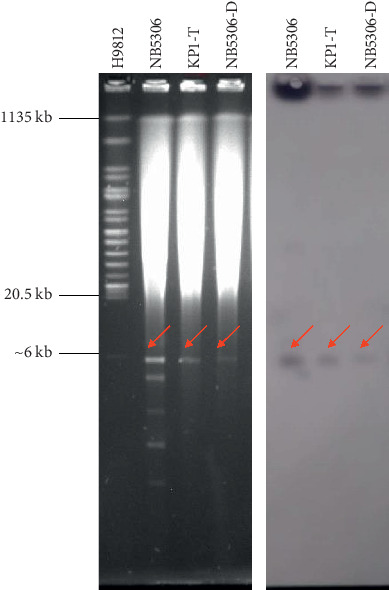
S1-PFGE and Southern blot of isolates. H9812: *Salmonella* H9812 molecular marker; NB5306: *K. pneumoniae* NB5306; KP1-T: DH5*α* electrotransformant of *K. pneumoniae* KP1; NB5306-D: DH5*α* chemical transformant of *K. pneumoniae* NB5306.

S1 PFGE and Southern blot showed that *bla*_OXA-232_ gene was located on ∼6 kb plasmid (Figure 1), and *bla*_OXA-232_ gene was located on a 6141 bp plasmid by primer walking sequencing.

The plasmid differs from the plasmid (GenBank accession no. KY454616) found in Shanghai with one nucleotide substitution [2], which contained nine open reading frames (MobA, MobB, MobD, ΔMobC, ΔISEcp1, blaOXA-232, ΔLysR, ΔEreA, and RepA). Plasmid finder revealed the *bla*_OXA-232_ gene was on a ColKP3-type plasmid.

## 4. Discussion

The OXA-232 carbapenemase-producing strains emerging had been frequently reported to have association with travelling to India. For example, Potron et al. reported that two *K. pneumoniae* and one *E. coli* harboring OXA-232 were recovered from three patients transferred from India to France in 2011 [7]. Findlay et al. reported that OXA-232 was found in isolates from patients reporting travel to India across the UK between 2007 and 2014 [10]. Jeong et al. reported that clonal and horizontal spread of the *bla*_OXA-232_ gene among *Enterobacteriaceae* in a Korean hospital was attributed to an index patient who was likely colonized during a prior hospitalization in India [11]. But some reported OXA-232 strains were not strongly relevant to the history of recent travel abroad. For example, Mancini et al. reported that 6 *K. pneumoniae* were recovered in 2017 from 5 different patients, who reported no recent travel abroad [12]. Abdul Momin et al. reported that 5 OXA-232-producing *K. pneumoniae* isolates were recovered from 5 patients in Brunei; all patients were hospitalized locally and had no history of recent travel [13]. In this study, the patient was hospitalized locally and reported no travel abroad history.

Previously reports showed that the dominant epidemic ST in *bla*_OXA-232_*K. pneumoniae* belonged to ST14 (France [7], the United States [14, 15], South Korea [11], and Middle East [16]), ST15 (China [2, 3] and Czech Republic [17]), ST16 (USA [14], Italy [18], and UK [10]), ST147 (India [19], Tunisia [20], and UK [10]), ST231 (Switzerland [12], the UK [10], Poland [21], Singapore [22], and Brunei Darussalam [13]), and ST395 (India [19] and UK [10]). Moreover, ST11 (India [19]) and hypervirulent ST23 (India [23]) OXA-232-producing *K. pneumoniae* have also been sporadically identified. In this research, we identified an OXA-232-producing strain belonging to ST437. ST437 belonged to clonal complex 11 (CC11), which was together with its single-locus variants ST11 and ST258 [24]. The high-risk multidrug-resistant CC11 is widespread worldwide, and cases with the CC11 strain faced more severe forms of drug resistance and treatment challenges than other clonal complex [8], especially KPC-producing and NDM-producing CC11. Among CC11, ST258 CRKP predominated in North America and Europe [25]; furthermore, ST11 was the primary CRKP clone in eastern Asia, especially in China [1]. What interested us was that we identified an OXA-232-producing CC11 isolates in China, which indicated that the OXA-48-type carbapenemase was spreading in high-risk CC11, like KPC and NDM.

*bla*_OXA-232_ *K. pneumoniae* was found in France [7] and China [2], and this study remained susceptible to colistin and tigecycline, but NB5306 showed different drug resistance profiles to carbapenems compared with strain RAN, which was the first *K. pneumoniae* strain harboring *bla*_OXA-232_ [7], with NB5306 MICs of ertapenem was 8 mg/L, imipenem was 0.25 mg/L, and meropenem was 2 mg/L, while RAN was resistant to ertapenem, imipenem, and meropenem, with MIC >32 mg/L [7]. NB5306-D obtained *bla*_OXA-232_ by chemical transformation, and the MIC of ertapenem was 1 mg/L, in which we conjectured that the *omp*K36 variant might play a role in charge of MIC of ertapenem [26].

Among studies involving OXA-232, only Jeong et al. reported that the *bla*_OXA-232_ gene was located on a conjugative ColE-type plasmid 6141 bp in size, and this plasmid was successfully transferred from *K. pneumoniae* H16 to *E. coli* J53 by conjugation [11]. However, this investigation and other studies reported that *bla*_OXA-232_ gene was located on a nonconjugative ColKP3-type plasmid 6141 bp in size [3, 7, 22]. Nonconjugative plasmids are incapable of initiating conjugation; however, they can be transferred with the assistance of conjugative plasmids. The same kind of 6 kb plasmids harboring *bla*_OXA-232_ were also found in Europe, North America, Asia, and North Africa [20]. It is speculated that 6 kb plasmid is likely disseminated in the whole world.

The study provides the emergence of an OXA-232 *K. pneumoniae* belonging to CC11. Further surveillance and investigations are needed to have better understanding of potential transmission and evolution of OXA-232 carbapenemase-producing CRKP in the world.

## Figures and Tables

**Table 1:** *In vitro* activities of antimicrobial agents against *K. pneumoniae* NB5306 and their transformants.

	Strain	NB5306	NB5306-D	*E. coli* DH5*α*	KP1-T [8]	KP1 [8]
	*β*-Lactamase (s)	OXA-232, TEM-1B, SHV-11, and CTX-M-55	OXA-232	OXA-232	OXA-232, SHV-1, and CTX-M-15
MIC (mg/L)	Ertapenem	8	1	0.03	0.25	32
	Meropenem	2	0.12	0.03	0.12	4
	Imipenem	0.25	0.5	0.06	2	1
	Cefotaxime	128				
	Ceftriaxone	256				
	Piperacillin/tazobactam	256				
	Amikacin	256				
	Gentamicin	256				
	Tobramycin	32				
	Ciprofloxacin	32				
	Levofloxacin	32				
	Fosfomycin	8				
	Colistin	0.03				
	Tigecycline	0.25				

NB5306: *K. pneumonia* NB5306; NB5306-D: DH5*α* chemical transformant of NB5306; KP1-T: DH5*α* electrotransformant of *K. pneumonia* KP1; KP1 = *K. pneumonia* KP1.

## Data Availability

The draft genome sequence of K. pneumoniae NB5306 and the complete nucleotide sequence of the plasmid carrying blaOXA-232 have been deposited in GenBank under accession numbers QYCO00000000 and MK105834, respectively.
